# Genome Sequence of the Potato Plant Pathogen *Dickeya dianthicola* Strain RNS04.9

**DOI:** 10.1128/genomeA.00581-15

**Published:** 2015-06-04

**Authors:** Yannick Raoul des Essarts, Samuel Mondy, Valérie Hélias, Denis Faure

**Affiliations:** a Institute for Integrative Biology of the Cell (I2BC), CNRS CEA University Paris-Sud, Saclay Plant Sciences, Gif-sur-Yvette, France; b Fédération Nationale des Producteurs de Plants de Pomme de Terre–Recherche, Développement, Promotion du Plant de Pommes de Terre (FN3PT-RD3PT), Paris, France

## Abstract

*Dickeya dianthicola* is one of the causative agents of soft rot and blackleg diseases, which are currently identified in European countries in a wide range of crops. Here, we report the draft genome sequence of *D. dianthicola* strain RNS04.9, which was isolated from a potato plant with blackleg symptoms in 2004.

## GENOME ANNOUNCEMENT

Pectinolytic bacteria of the genera *Pectobacterium* and *Dickeya* are causative agents of soft rot and blackleg diseases that affect a wide range of field crops and ornamental plants (1–5). In Europe, *Pectobacterium atrosepticum, Pectobacterium carotovorum* subsp. *carotovorum, Dickeya dianthicola*, and *Dickeya solani* are the main pectinolytic pathogens of potato in the field stage or during storage (6–8). Two *Dickeya* species emerged recently: *D. dianthicola* in the 1970s and *D. solani* in the 2000s (9). The pathogens may propagate from tubers to stems or leaves and reciprocally from aerial parts to progeny tubers (10–13).

Our team has already reported the genome sequences of other blackleg and soft rot diseases agents, *P. atrosepticum* strain CFBP6276 (14) and *D. solani* strain PRI3337 (15, 16). *D. dianthicola* strain RNS04.9 was isolated and characterized in 2004 from a potato plant with blackleg symptom sampled in a French field during the epidemiological annual survey monitored by the FN3PT/RD3PT. This strain has been investigated in several former studies (17–19) and used to develop a valuable greenhouse pathosystem, which allowed the assessment of a biocontrol strategy (20).

Genomic DNA of *D. dianthicola* RNS04.9 was subjected to next-generation Illumina HiSeq 2000 version 3 technology. Two libraries were constructed: a paired-end library with a fragment size of 150 to 500 bp and a shotgun long-jumping-distance mate-pair library with an insert size of 8,000 bp. Sequencing of the libraries was carried out using a 2 × 100 bp paired-end read module by Eurofins Genomics. Assembly was performed by CLC Genomics Workbench version 5.5 (from CLCbio). Sequence reads were trimmed based on quality (threshold 0.05), and minimum size (above 60 nucleotides). Scaffolding of the contig was processed using SSPACE basic version 2.0 (21). A total of 46 contigs were generated by *de novo* assembly and clustered in 2 scaffolds. The first scaffold (3,366,760 bp) comprised 35 contigs, the 11 remaining ones being included in the second scaffold (1,343,990 bp). The *in silico* closure of some gaps was carried out by mapping (read length of 0.9 and similarity of 0.95) the mate-pair reads on each of the 5-kbp contig ends. Following this treatment, 36 gaps were elucidated. Then, the collected reads were used for *de novo* local assembling (read length of 0.5 and similarity of 0.8). This allowed the generation of one scaffold with 10 gaps, from 28 bp to 3,271 bp in size. Some gaps were resolved by Sanger sequencing of PCR amplicons.

At the end, the genome sequence of *D. dianthicola* strain RNS04.9 revealed a circular chromosome consisting of 4,721,506 bp and comprised 7 contigs. No plasmid was detected. The G+C content was ~56%. Gene prediction using the RAST version 4.0 automated pipeline (22, 23) revealed the presence of 4,351 coding sequences and 97 RNA-assimilated regions. These features are in accordance with other data of the *Dickeya* spp. genomes and draft genomes that are available in public databases.

### Nucleotide sequence accession numbers.

This draft genome sequence has been deposited at DDBJ/EMBL/GenBank under the accession number APVF00000000. The version described in this paper is the first version, APVF01000000.

## References

[1] Gardan L, Gouy C, Christen R, Samson R 2003 Elevation of three subspecies of *Pectobacterium carotovorum* to species level: *Pectobacterium atrosepticum* sp. nov., *Pectobacterium betavasculorum* sp. nov. and *Pectobacterium wasabiae* sp. nov. Int J Syst Evol Microbiol 53:381–391. doi:10.1099/ijs.0.02423-0. 12710602

[2] Ma B, Hibbing ME, Kim H-S, Reedy RM, Yedidia I, Breuer J, Breuer J, Glasner JD, Perna NT, Kelman A, Charkowski AO 2007 Host range and molecular phylogenies of the soft rot enterobacterial genera *Pectobacterium* and *Dickeya*. Phytopathology 97:1150–1163. doi:10.1094/PHYTO-97-9-1150. 18944180

[3] Perombelon MCM, Kelman A 1980 Ecology of the soft rot erwinias. Annu Rev Phytopathol 18:361–387. doi:10.1146/annurev.py.18.090180.002045.

[4] Pérombelon MCM 1992 Potato blackleg: epidemiology, host-pathogen interaction and control. Neth J Plant Pathol 98:135–146. doi:10.1007/BF01974480.

[5] Samson R, Legendre JB, Christen R, Fischer-Le Saux M, Achouak W, Gardan L 2005 Transfer of *Pectobacterium chrysanthemi* (Burkholder et al. 1953) Brenner et al. 1973 and *Brenneria paradisiaca* to the genus *Dickeya* gen. nov. as *Dickeya chrysanthemi* comb. nov. and *Dickeya paradisiaca* comb. nov. and delineation of four novel species, *Dickeya dadantii* sp. nov., *Dickeya dianthicola* sp. nov., *Dickeya dieffenbachiae* sp. nov. and *Dickeya zeae* sp. nov. Int J Syst Evol Microbiol 55:1415–1427. doi:10.1099/ijs.0.02791-0. 16014461

[6] Hauben L, Moore ER, Vauterin L, Steenackers M, Mergaert J, Verdonck L, Swings J 1998 Phylogenetic position of phytopathogens within the *Enterobacteriaceae*. Syst Appl Microbiol 21:384–397. doi:10.1016/S0723-2020(98)80048-9. 9779605

[7] Van der Wolf JM, Nijhuis EH, Kowalewska MJ, Saddler GS, Parkinson N, Elphinstone JG, Pritchard L, Toth IK, Lojkowska E, Potrykus M, Waleron M, de Vos P, Cleenwerck I, Pirhonen M, Garlant L, Hélias V, Pothier JF, Pflüger V, Duffy B, Tsror L, Manulis S 2014 *Dickeya solani* sp. nov., a pectinolytic plant-pathogenic bacterium isolated from potato (*Solanum tuberosum*). Int J Syst Evol Microbiol 64:768–774. doi:10.1099/ijs.0.052944-0. 24225027

[8] Van der Wolf JM, De Boer SH 2007 Bacterial pathogens of potato, p 595–617. *In* Vreugdenhil D, Bradshaw J, Gebhardt C, Govers F, Mackerron DKL, Taylor MA, Ross HA (ed), Potato biology and biotechnology. Elsevier, Amsterdam.

[9] Toth IK, van der Wolf JM, Saddler G, Lojkowska E, Hélias V, Pirhonen M, Tsror Lahkim L, Elphinstone JG 2011 *Dickeya* species: an emerging problem for potato production in Europe: *Dickeya* spp. on potato in Europe. Plant Pathol 60:385–399. doi:10.1111/j.1365-3059.2011.02427.x.

[10] Czajkowski R, Pérombelon MCM, van Veen JA, van der Wolf JM 2011 Control of blackleg and tuber soft rot of potato caused by *Pectobacterium* and *Dickeya* species: a review. Plant Pathol 60:999–1013. doi:10.1111/j.1365-3059.2011.02470.x.

[11] Czajkowski R, Grabe GJ, van der Wolf JM 2009 Distribution of *Dickeya* spp. and *Pectobacterium carotovorum* subsp. *carotovorum* in naturally infected seed potatoes. Eur J Plant Pathol 125:263–275. doi:10.1007/s10658-009-9480-9.

[12] Czajkowski R, de Boer WJ, van Veen JA, van der Wolf JM 2010 Downward vascular translocation of a green fluorescent protein-tagged strain of *Dickeya* sp.(biovar 3) from stem and leaf inoculation sites on potato. Phytopathology 100:1128–1137. doi:10.1094/PHYTO-03-10-0093. 20932162

[13] Czajkowski R, de Boer WJ, Velvis H, van der Wolf JM 2010 Systemic colonization of potato plants by a soilborne, green fluorescent protein-tagged strain of *Dickeya* sp. biovar 3. Phytopathology 100:134–142. doi:10.1094/PHYTO-100-2-0134. 20055647

[14] Kwasiborski A, Mondy S, Beury-Cirou A, Faure D 2013 Genome sequence of the *Pectobacterium atrosepticum* strain cfbp6276, causing blackleg and soft rot diseases on potato plants and tubers. Genome Announc 1(3):e00374-13. doi:10.1128/genomeA.00374-13. PMC3707594 23788545

[15] Khayi S, Mondy S, Beury-Cirou A, Moumni M, Helias V, Faure D 2014 Genome sequence of the emerging plant pathogen *Dickeya solani* strain RNS 08.23.3.1A. Genome Announc 2(1):e01270-13. doi:10.1128/genomeA.01270-13. PMC3907742 24482527

[16] Pédron J, Mondy S, des Essarts YR, Van Gijsegem F, Faure D 2014 Genomic and metabolic comparison with *Dickeya dadantii* 3937 reveals the emerging *Dickeya solani* potato pathogen to display distinctive metabolic activities and T5SS/T6SS-related toxin repertoire. BMC Genomics 15:283. doi:10.1186/1471-2164-15-283. 24735398 PMC4028081

[17] Hélias V, Hamon P, Huchet E, Wolf JVD, Andrivon D 2012 Two new effective semiselective crystal violet pectate media for isolation of *Pectobacterium* and *Dickeya*. Plant Pathol 61:339–345. doi:10.1111/j.1365-3059.2011.02508.x.

[18] Crépin A, Barbey C, Beury-Cirou A, Hélias V, Taupin L, Reverchon S, Nasser W, Faure D, Dufour A, Orange N, Feuilloley M, Heurlier K, Burini J-F, Latour X 2012 Quorum sensing signaling molecules produced by reference and emerging soft-rot bacteria (*Dickeya* and *Pectobacterium* spp.). PLoS One 7:e35176. doi:10.1371/journal.pone.0035176. 22539957 PMC3335102

[19] Crépin A, Beury-Cirou A, Barbey C, Farmer C, Hélias V, Burini J-F, Faure D, Latour X 2012 *N*-Acyl homoserine lactones in diverse *Pectobacterium* and *Dickeya* plant pathogens: diversity, abundance, and involvement in virulence. Sensors 12:3484–3497. doi:10.3390/s120303484. 22737020 PMC3376598

[20] Cigna J, Raoul des Essarts Y, Mondy S, Hélias V, Beury-Cirou A, Faure D 2015 Draft genome sequences of *Pseudomonas fluorescens* strains PA4C2 and PA3G8 and *Pseudomonas putida* PA14H7, three biocontrol bacteria against *Dickeya* phytopathogens. Genome Announc 3(1):e01503–14. doi:10.1128/genomeA.01503-14. PMC4319517 25635023

[21] Boetzer M, Henkel CV, Jansen HJ, Butler D, Pirovano W 2011 Scaffolding pre-assembled contigs using SSPACE. Bioinformatics 27:578–579. doi:10.1093/bioinformatics/btq683. 21149342

[22] Aziz RK, Bartels D, Best AA, DeJongh M, Disz T, Edwards RA, Formsma K, Gerdes S, Glass EM, Kubal M, Meyer F, Olsen GJ, Olson R, Osterman AL, Overbeek RA, McNeil LK, Paarmann D, Paczian T, Parrello B, Pusch GD, Reich C, Stevens R, Vassieva O, Vonstein V, Wilke A, Zagnitko O 2008 The RAST server: Rapid Annotations using Subsystems Technology. BMC Genomics 9:75. doi:10.1186/1471-2164-9-75. 18261238 PMC2265698

[23] Overbeek R, Olson R, Pusch GD, Olsen GJ, Davis JJ, Disz T, Edwards RA, Gerdes S, Parrello B, Shukla M, Vonstein V, Wattam AR, Xia F, Stevens R 2014 The SEED and the rapid annotation of microbial genomes using subsystems technology (RAST). Nucleic Acids Res 42:D206–D214. doi:10.1093/nar/gkt1226. 24293654 PMC3965101

